# Genome Sequence of the Caproic Acid-Producing Bacterium Caproiciproducens galactitolivorans BS-1^T^ (JCM 30532)

**DOI:** 10.1128/MRA.00346-19

**Published:** 2019-08-01

**Authors:** Frank R. Bengelsdorf, Anja Poehlein, Rolf Daniel, Peter Dürre

**Affiliations:** a Institut für Mikrobiologie und Biotechnologie, Universität Ulm, Ulm, Germany; b Genomic and Applied Microbiology & Göttingen Genomics Laboratory, Georg-August University Göttingen, Göttingen, Germany; University of Southern California

## Abstract

Caproiciproducens galactitolivorans BS-1^T^ is an anaerobic bacterium that produces acetate, butyrate, and caproate. The genome has a size of 2.57 Mbp and harbors 2,439 predicted protein-coding genes.

## ANNOUNCEMENT

Caproiciproducens galactitolivorans BS-1^T^, a non-spore-forming, anaerobic, Gram-positive bacterium, was isolated from an anaerobic digestion reactor (1). Strain BS-1^T^ is able to produce acetic, butyric, and caproic acid using, e.g., the sugar alcohol d-galactitol as the substrate (2). In *in situ* extractive fermentations, strain BS-1^T^ produced 6.96 g liter^−1^ hexanoic acid from galactitol, with a maximum rate of 0.34 g liter^−1^ h^−1^ (3). Other validly described bacterial strains producing caproate are, namely, Clostridium carboxidivorans (4), Clostridium kluyveri (5), Eubacterium limosum (6), Eubacterium pyruvativorans (7), Megasphaera elsdenii (8), Megasphaera cerevisiae (9), and Rhodospirillum rubrum (10). The genome of strain BS-1^T^ was sequenced to compare its genetic features with those of other caproate-producing strains.

Cells of strain BS-1^T^ were grown as described by Kim et al. (1), and DNA was extracted using the MasterPure Gram-positive DNA purification kit (Lucigen Corporation). DNA of strain BS-1^T^ was used to generate Illumina shotgun paired-end sequencing libraries, which were sequenced with a MiSeq instrument and the MiSeq reagent kit version 3 (600 cycles), as recommended by the manufacturer (Illumina, San Diego, CA, USA), resulting in 970,997 paired-end reads. Quality filtering using Trimmomatic version 0.36 (-phred33, ILLUMINACLIP:2:30:10, LEADING:3 TRAILING:3 SLIDINGWINDOW:4:15 MINLEN:50) (11) resulted in 1,885,280 paired-end reads with an average read length of 225 bp. The assembly was performed with the SPAdes genome assembler software version 3.9.0, with default settings (12). The assembly resulted in 33 contigs (>500 bp) and an average coverage of 164-fold. The assembly was validated, and the read coverage was determined with Qualimap version 2.1 (13) with default settings. The draft genome of strain BS-1^T^ consists of 33 contigs (2.57 Mbp), with an overall GC content of 48.1%. Automatic gene prediction and identification of rRNA and tRNA genes were performed using the software tool Prokka (14) with default settings. The draft genome contained 5 rRNA genes, 52 tRNA genes, 1,925 protein-coding genes with predicted functions, and 549 genes coding for hypothetical proteins.

The genome analysis of strain BS-1^T^ showed the presence of genes encoding the *bcs* operon (butyryl-coenzyme A [butyryl-CoA] synthesis) as initially described for Clostridium acetobutylicum (15). In strain BS-1^T^, the gene encoding the thiolase is additionally found in the respective gene cluster. The genome comprises a complete gene cluster encoding the Rnf (*Rhodobacter* nitrogen fixation) complex. This protein complex oxidizes reduced ferredoxin (Fd^2−^) and yields NADH while generating an ion gradient (protons or Na^+^) across the cytoplasmic membrane (16, 17); it therefore functions as a ferredoxin:NAD^+^ oxidoreductase (18). For *C. kluyveri,* it was assumed that the Fd^2−^ generated during crotonyl-CoA reduction (butyryl-CoA dehydrogenase coupled to electron transport flavoproteins [Bcd-Etf complex]) is used for the regeneration of NADH via the Rnf complex (19). It is likely that in strain BS-1 the Rnf complex also functions as a ferredoxin:NAD^+^ oxidoreductase to oxidize Fd^2−^ formed by the pyruvate:ferredoxin oxidoreductase or by the Bcd-Etf complex.

### Data availability.

This whole-genome shotgun project has been deposited at DDBJ/ENA/GenBank under the accession number SRMQ00000000. The version described in this paper is version SRMQ01000000. Raw data have been deposited at the NCBI SRA database under the accession number SRR9077739.
